# Serum anti-P53 antibodies and alpha-fetoprotein in patients with non-B non-C hepatocellular carcinoma

**DOI:** 10.1186/2193-1801-2-69

**Published:** 2013-02-25

**Authors:** Abdel Raouf Abou El Azm, Mohamed Yousef, Raafat Salah, Wael Mayah, Salwa Tawfeek, Hussien Ghorabah, Nagwa Mansour

**Affiliations:** 1 Faculty of Medicine, Egypt and president of the Egyptian Society of Liver and Environment, Tanta University, Tanta, Egypt; 2 Faculty of Medicine, Tanta University, Tanta, Egypt; 3 National research Institute, Cairo, Egypt

**Keywords:** Hepatocellular carcinoma, Non-B non-C HCC, Hepatitis C, P53 antibodies, Alpha-fetoprotein

## Abstract

The rate of hepatocellular carcinoma (HCC) is increasing worldwide including Egypt. Non-B non-C HCC was reported in some countries. We aimed to investigate P53 antibodies and alpha-fetoprotein in patients with non-B non-C HCC in our region. In a case series study, included 281 patients with HCC and 20 patients with liver cirrhosis of matched age, sex and social factors were received for management at Tanta University Hospitals. Sera were tested for HCV and HBV markers by ELISA/PCR, alpha-fetoprotein (AFP) level and anti-p53 antibody were evaluated by ELISA. Antinuclear antibody, serum copper and iron were assessed in non-viral HCC. Liver scanning and biopsy were evaluated. Non-B non-C HCC patients were 13.87% of total. P53 antibody serum level in non-B non-C HCC patients showed insignificant difference (p>0.05) as compared to viral-associated HCC, while significant as compared to cirrhosis. They had significant decrease in serum AFP level (p<0.001) as compared to viral-associated HCC. Their tumors were mainly solitary, and have smaller-sizes. Sensitivity, specificity, PPV, NPV and accuracy test of anti P53 antibody positive patients were 91.52%, 84.63%, 90.34%, 80.2% and 74.8% respectively. It correlates positively with AFP, tumor size and staging, MELD score and Child-Pugh score.

Non-B non-C HCC showed high serum prevalence of anti-p53 as viral-associated HCC suggesting an evidence of high onchogenecity. It appears of much benefit in diagnosis, follow up and differentiation from cirrhosis in presence of low levels of alpha-fetoprotein.

## Introduction

There is a heterogeneous distribution of HCC at regional and international levels due to infectious and/or environmental factors that may contribute to risk (Lehman et al. 2007). Egypt has the highest prevalence of HCV worldwide and has rising rates of HCC (Lehman and Wilson 2009).

The major risk factors include chronic HBV and HCV infections and chemical exposures (Wang et al. 2002, and Ertle et al. 2010). Recently, the proportion of non-B non-C HCC has been increasing in many areas of the world (Ertle et al. 2010). The pattern of HCC and its risk factors is changing (Anwar et al. 2008).

The p53 protein is involved in DNA repair and is an oncoprotective antigen. This gene when damaged, leads to production of anti-p53 and predisposes to various cancers, including HCC (Di Cesare et al. 2001 , and Ndububa et al. 2001).

P53 antibodies are predominantly associated with p53 gene mutations (Soussi 2000) in the sera of patients with various types of cancer (Shimada et al. 2003). It was reported with high titration in viral-associated HCC Egyptian patients (Atta et al. 2008).

Due to the alarming increase in the incidence of HCC, there is a need for recent insights on contribution of emerging risk factors of hepatocellular carcinogenesis (Abdel-Hamid 2009), and provide more effective measures for early diagnosis, monitor progress and intervention.

We aimed to determine prevalence and diagnostic utility of P53 antibodies and alpha fetoprotein in patients with non-B non-C HCC in our region.

## Patients and methods

### Patients

In a case series retrospective study, included 281 patients with HCC were received for management at Tanta University Hospitals, within the last 3 years (from March 2009 to February 2012) and twenty patients with cirrhosis, and without any evidence of HCC as control, with matched age, sex, and social factors.

### Study area

(Gharbia Governorate): The residence area of patients is generally a rural area containing villages and districts; most people are working in agriculture, and the area is highly endemic with HCV, and to a lesser extent HBV. This area has important chemical industrial factories in Kafr El-Zayat district, where unfortunately non-B non-C HCC patients were received.

### Methods

All patients were subjected to: History taking included demographic variables, and environmental exposures. Clinical assessment and diagnosis of HCC was based on detection of hepatic focal lesions by imaging techniques (ultrasonography with, or without triphasic CT scans) plus serum alpha-fetoprotein (Sorin Biomedica - 3rd generation ELISA) and guided liver & tumor biopsy for histopathological confirmation. Tumor staging was done according to Llovet, et al. (1999).

### Serological investigations for viral etiology

Sera were tested for HCV antibody (Qualitest HCV-3rd generation ELISA) confirmed by RT-PCR using the automated Cobas Amplicor system of Roche. HBV infection was assessed using: HBs Ag and HBc Ab IgG (in negative HBs Ag) by 3^rd^ generation ELISA, and HBV-DNA for patients with negative HBs Ag and HBc Ab IgG using the automated Cobas Amplicor system of Roche.

Serum total iron binding capacity, ceruloplasmin, and antinuclear antibody, were evaluated for the etiology of HCC patients.

Anti-p53 antibodies were evaluated by a modified ELISA test (Atta et al. 2008) to estimate the levels in sera of HCC patients and control groups, as a modification of Engvall and Perlmann (1971).

### The procedure in brief is as follows

Polystyrene microtiter plates (Nunc Maxisorp, flat bottom) were coated with 100 μL of recombinant wild-type human p53 protein (Sigma Chemical Company, USA) in the concentration of 5 μg/mL in Carbonate buffer, 0.06 M, pH 9.6. The coated plate was incubated overnight at room temperature under humidified atmosphere. The plates were washed four times with phosphate buffered saline (PBS) containing 0.1% Tween 20 (PBS-T). The non-specific sites in the wells were blocked with 0.2% non-fat milk for 2 hours at 37°C. After 4 washes with PBS-T, the plates were incubated with 1:1000 dilutions of the sera from patients and control groups. After 2 hours incubation at 37°C and washing, anti-human IgG whole molecule alkaline phosphatase conjugate (Sigma Chemical company, USA), at dilution 1:500 in PBS-T containing 0.2% non-fat milk, was added as the secondary antibody. At the end of 2 hours incubation at 37°C and washing, the color was developed by the addition of 100 μL of the substrate, para-nitrophenyl phosphate (Sigma Chemical Company, USA) to each well. After arresting the reaction with 50 μL of 3 N HCl, the optical density reading was taken in the microplate spectrophotometer (EL311 microplate autoreader, Bio-Tek instruments, USA) at 405 nm wave length. Cutoff level of ELISA above or below which the tested samples were considered positive or negative was calculated as the mean concentration using 0.04 OD cutoff points.

The study was approved by the Ethical and Research Committee of Tanta Faculty of Medicine and an informed consent was taken from each participant.

### Statistical analysis

Was performed by the statistical software SPSS 11 using independent-sample *t* test. Chi-square was applied for the results. P < 0.05 was considered statistically significant, and < 0.001 highly significant. Analysis of variance [ANOVA] tests by SPSS V.16. Linear Correlation Coefficient [r] of the results was carried out.

## Results

Serum total iron binding capacity, ceruloplasmin, and antinuclear antibody were detected in average values in non-B non-C HCC patients.

Table 1 showed: Non-B non-C ratio in HCC patients were 39/281 = 13.87%, HCV were 186/281 =66.19%, HBV were 26/281 = 9.25% and HCV/HBV co-infection 29/281 = 10.32%. There is a rising incidence per year without significant difference P > 0.05.

**Table 1:** **Number of viral and non-viral HCC patients and ratios/ year**

Number in Years	Viral (HCV, HBV and co-infect.)	Non-B non-C	Total
- First year	74 (C: 57, B: 10, C&B: 7) =30.58%	5 =12.82%	79 =28.11%
- Second year	83 (C: 62, B: 10, C&B:11) =34.30%	10 =25.64%	93 =33.10%
- Third year	85 (C: 67, B: 6, C&B:12) =35.12%	24 = 61.54%	109 =37.79%
Total in 3 years	242 (C: 186, B: 26, C&B:30) = 100%	39 = 100%	281 = 100%

There is insignificant increase in number of total HCC, viral, non-viral HCC patients when comparing between first, second and third year (P-Value >0.05).

Table 2 showed: The frequency of anti-p53 antibodies using a cutoff point of 0.4 OD in viral-associated HCC patients, were positive in 168 of 242 (69.42%), non-B non-C HCC in 26 of 39 (66.66%) and liver cirrhosis in 4 of 20 patients (20%). No significant difference was detected between all groups as regard to age, and six (p > 0.05). Serum level of P53 antibodies in non-B non-C HCC patients showed insignificant difference (p > 0.05) as compared to viral-associated HCC, while significant as compared to cirrhosis. They had significant decrease in serum alpha-fetoprotein level (p < 0.001) as compared to viral-associated HCC. Their tumors were mainly solitary and have smaller-sizes.

**Table 2:** **Liver grading, tumors features, AFP and anti P53 findings in patient groups**

Items	Viral (n = 242)	Non-B nonC (n = 39)	Cirrhosis (n = 20)	P1	P2	P3
**Age (years)**	51.9 ± 11.7	55.5 ± 6.2	53.1 ± 95	>0.05	>0.05	>0.05
**Gender:**						
- Male	185(76.45%)	37(94.87%)	15(75.00%)	>0.05	>0.05	>0.05
- Female	57(23.55%)	2(05.13%)	4(20.00%)	>0.05	>0.05	>0.05
**Severity of liver disease: Child-Pugh score**						
- Class A	80(33.06%)	24(61.54%)	4(20.00%)	< 0.05*	>0.05	>0.05
- Class B	48(19.83%)	9(23.08%)	7(35.00%)	>0.05	>0.05	>0.05
- Class C	114(47.11%)	6(15.38%)	9(45.00%)	< 0.05*	>0.05	>0.05
**(MELD score)**						
- Early (6-11)	48(19.83%)	23(58.97%)	3(15.00%)	<0.001*	>0.05	<0.05*
- Intermed.(12-18)	84(34.71%)	10(25.64%)	8(40.00%)	>0.05	>0.05	>0.05
- Sever (19-40)	110(45.45%)	6(15.38%)	9(55.00%)	< 0.05*	>0.05	>0.05
**Anti P53 antibody + ve**	168(69.42%)	26(66.66%)	4(20.00%)	>0.05	< 0.05*	<0.05*
**AFP (ng/mL)**						
- <200	52(21.48%)	31(79.49%)	20(100%)	<0.001*	<0.001*	>0.05
- 200-500	84(34.71%)	5(12.82%)	**-**	>0.05	< 0.05*	>0.05
- >500	106(43.80%)	3(07.69%)	**-**	< 0.05*	< 0.05*	>0.05
**Tumor findings US/CT**						
**Size (cm):**						
< 3	44(18.18%)	26(66.66%)	**-**	<0.001*	**-**	**-**
3-5	98(40.50%)	9(23.08%)	**-**	>0.05	**-**	**-**
>5	100(41.32%)	4(10.26%)	**-**	< 0.05*	**-**	**-**
**Number of tumors:**						
Single	148(61.16%)	33(84.62%)	**-**	>0.05	**-**	**-**
Multiple	94(38.84%)	6(15.38%)	**-**	>0.05	**-**	**-**

Significant* P value < 0.05 Highly Significant * P value < 0.001.

P1 = viral vs. non-viral group P2 = viral vs. cirrhosis group.

P3 = non-viral vs. cirrhosis group.

### Severity of liver disease

#### Child-Pugh score

- Class A (*X*^2^ 1 = 11.69, *X*^2^ 2 = 1.45, *X*^2^ 3 = 9.15). There was a significant increase in non-B non-C HCC group when compared to viral group.
- Class B (*X*^2^ 1 = 0.22, *X*^2^ 2 = 2.56, *X*^2^ 3 = 0.95)
- Class C (*X*^2^ 1 = 13.8, *X*^2^ 2 = 0.033, X^2^3 = 6.12). There was a significant increase in viral group when compared to non-B non-C group.

Table 3: showed: Sensitivity, specificity, PPV, NPV and accuracy test of anti P53 antibody positive patients were 91.52%, 84.63%, 90.34%, 80.2% and 74.8% respectively.

**Table 3:** **Sensitivity, Specificity, PPV, NPV and Accuracy test of anti P53 antibody + ve patients**

	Anti P53 antibody + ve
**Sensitivity**	**91.52%**
**Specificity**	**84.63%**
**PPV**	**90.34%**
**NPV**	**80.2%**
**Accuracy**	**74.8%**

Table 4: showed significantly positive correlations of P53 antibody with AFP, tumor size, tumor number, MELD score, Child-Pugh score, and Tumor staging.

**Table 4:** **Correlations of P-53 antibody with AFP, tumor size, tumor number, MELD score, Child-Pugh score, and Tumor staging**

P-53 antibody	Non-B non-C		Viral-associated	
	r.	p. value	r.	p. value
**AFP (ng/mL)**	**0.704**	**0.001**	**0.880**	**0.001**
**Tumor Size (cm)**	**0.829**	**0.001**	**0.896**	**0.001**
**Tumor Number**	**0.573**	**0.001**	**0.815**	**0.001**
**MELD score**	**0.790**	**0.001**	**0.848**	**0.001**
**Child-Pugh score**	**0.764**	**0.001**	**0.810**	**0.001**
**Tumor stage**	**0.856**	**0.01**	**0.472**	**0.003**

**Figure 1:**
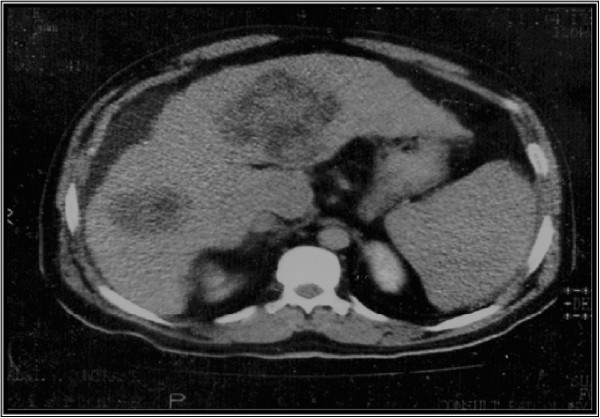
**Showing CT scan with HCC of variant size in both lobes of liver.**

**Figure 2:**
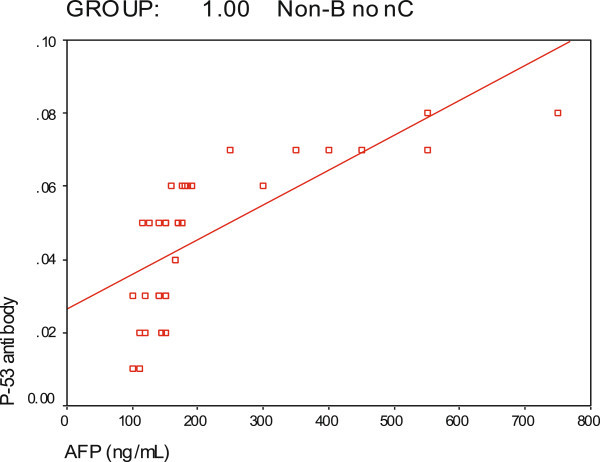
**Correlation of P-53 antibody with alpha fetoprotein in non-B non-C HCC group.**

**Figure 3:**
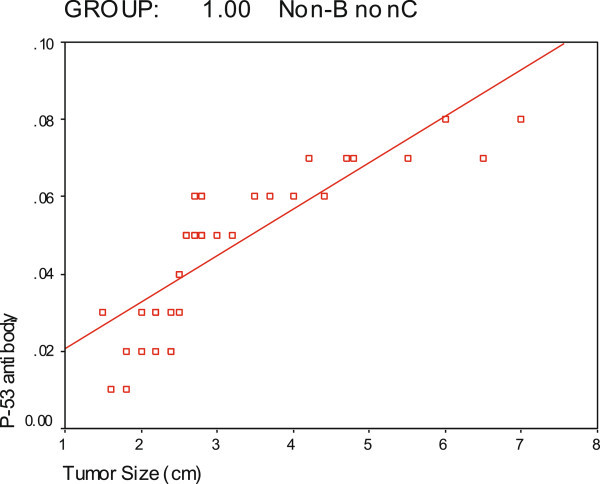
**Correlation of P-53 antibody with tumor size in non-B non-C HCC group.**

Figure 1: showed CT scan with HCC in both lobes of liver of variant size while Figures 2 and 3 showed positive correlations of P-53 antibody with alpha fetoprotein in non-B non-C HCC group and P53 antibody with tumor size in non-B non-C HCC respectively.

## Discussion

Chronic HBV, and HCV, are the most important risk factors in the development of HCC (Tornai 2010) in agreement with the present results. Egypt has the highest prevalence of HCV worldwide, and has rising rates of HCC (Lehman and Wilson 2009). Prevalence of HBV, and HCV were reported 25.9%, and 78.5% among HCC cases respectively (Severi et al. 2010).

HCC in previous studies confirmed wide international variation risks (Franceschi and Raza 2009). Multiple non-viral factors have been implicated in the development of HCC (Soliman et al. 2010). Approximately, 10% of HCC patients were reported negative for both HBV markers and antibodies to HCV (Kusakabe et al. 2007). In the current work, a higher rate 13.87% of non-B non-C was detected, while Abe et al. (2008) reported increasing ratio from 17.8% in 2000 to 28.6% in 2006 in Japan. This difference in prevalence, between Egypt, and Japan could be attributed to the difference in environmental risks, and the higher prevalence of HCV in Egypt.

In the current study, no significant difference was detected between non-B non-C HCC, and those of viral-association, as regard to age, or gender in agreement with previous reports (Asahina et al. 2010, and Yeh and Chen 2010). Some difference could be attributed to the nature of work making men more exposed to more risks, and/or the intensity of these risks.

According to our knowledge, there are no previous reports for non-B non-C HCC in our region, but reports of exposure to chemicals in HCV-associated HCC were reported. Chemicals can induce hepatic carcinogenesis through direct hepatotoxicity, inducing oxidative stress, and/or causing steatohepatitis (Angulo 2002), which seems to have a cumulative effect.

In our study, serum total iron binding capacity, ceruloplasmin, and antinuclear antibody were detected in average values in non-B non-C HCC patients. This could exclude the role of iron, copper and auto immunity in hepatic carcinogenesis of this group.

In the current study, anti-p53 showed insignificant difference between both HCC groups of patients, suggesting the presence of non-viral onchogens in non-B non-C HCC patients. This could agree with previous results, with high percentage of positivity of anti-p53 antibodies in Egyptian healthy subjects (Attallah et al. 2009, and Gadelhak et al. 2009). They mentioned that tumor suppressor genes may play a role in the puzzle of hepatic carcinogenesis. The finding of P53 antibodies in sera of individuals who are at high risk of cancer, as workers exposed to chemicals indicates that they have onchogenic potential, and promising in the early detection of cancer. Expressions were more pronounced in patients with HCC more than patients with liver cirrhosis, which could be of clinical importance for early diagnosis. This could be explained by interactions of chemical carcinogens, and genetic variations, are possible in HCC (Zhang 2010).

Alpha fetoprotein showed significantly lower levels in non-B non-C HCC, as compared to patients with viral-associated HCC in agreement with a previous report (Yamagishi et al. 2004), as tumors were detected of almost solitary, and of small sizes. High ratios of sensitivity, specificity, PPV, NPV and accuracy test of anti P53 antibody positive patients which could suggest clinical significance in non-B non-C HCC patients.

## Conclusion

The study revealed that HCC increasing rate is not only due to high endemicity of HCV and/or HBV but also due to non-B non-C environmental risks. Low serum alpha-fetoprotein level in non-B non-C HCC may add a difficulty in screening of these patients. The high prevalence of serum anti-p53 in our study could suggest evidence of high onchogenicity and could be of help in diagnosis and intervention in presence of low levels of alpha-fetoprotein.
